# Professional dog trainers’ perspectives on training methods: ethical and evidentiary insights

**DOI:** 10.3389/fvets.2026.1744448

**Published:** 2026-02-25

**Authors:** Jamie L. DeLeeuw, Todd J. Williams

**Affiliations:** 1 Community Research Plus, Grand Rapids, MI, United States; 2 Psychology Department, Grand Valley State University, Allendale, MI, United States

**Keywords:** animal welfare, applied ethics, aversive methods, dog training, epistemology, positive reinforcement, professional regulation, qualitative research

## Abstract

The professional dog training field sits at the intersection of applied behavioral science, ethics, and lived experience. Despite its significant animal welfare implications, it remains largely unregulated. This primarily qualitative study, complemented by quantitative analyses, examined how professional trainers with differing methodological orientations conceptualize humane and effective practice. Using stratified sampling, 35 trainers affiliated with independent certification directories (17 reward-based; 18 mixed methods) completed a pre-screen survey and semi-structured interviews. Data were analyzed to explore associations among training approach, certification, and demographics, as well as differences in ethical reasoning, evidentiary interpretation, and views on industry regulation. Across orientations, trainers consistently identified positive reinforcement as their most frequently used and effective method, expressed strong commitments to canine emotional well-being and owner education, and voiced concern over the industry’s lack of professional regulation. However, ethical and epistemic orientations diverged. While both groups evaluated methods in relation to canine welfare and behavioral outcomes, reward-based trainers more often grounded their practice in behavioral science and articulated deontological concerns regarding the intentional use of fear or pain. Mixed methods trainers more frequently employed consequentialist reasoning, supporting conditional use of aversive methods in specific contexts and placing comparatively greater emphasis on practitioner-based expertise when interpreting evidence. Although mixed methods trainers reported using positive reinforcement most often, they rated positive punishment and positive reinforcement as equally effective in independent assessments. Overall, the findings depict a profession characterized by ethical pluralism and epistemic tension, yet marked by sustained reflection and adaptive learning. To strengthen professional cohesion and enhance the practical relevance of future research and ethical frameworks, we recommend structured adversarial collaboration embedded within a community-based participatory research approach.

## 1 Introduction

Each year, millions of dogs enter the animal shelter system in the United States. In 2024, organizations participating in Shelter Animals Count (1) reported more than 2.9 million dog intakes, underscoring the scale of demand for rehoming and behavior support. Among these dogs, nearly a third (29%) were relinquished by their owners. Although socioeconomic and situational factors account for much of dog relinquishment, behavior problems also contribute in a meaningful way. Studies indicate that up to 40% of dog owners cite behavioral concerns—such as hyperactivity, aggression, or destructiveness—as reasons for surrender (2–7).

Behavior-related challenges can carry disproportionate welfare consequences, lending urgency to debates about how best to train dogs. Studies consistently show that dogs returned to shelters post-adoption often had owner-reported behavioral problems (3, 4, 8–10). Existing research on the topic, while limited, has found that dogs relinquished for behavioral reasons were less likely to be adopted and more likely to be euthanized than those surrendered for owner-related reasons such as finances (11, 12). Consequently, these findings demonstrate that training is not merely about teaching skills but about mitigating behaviors that carry significant welfare consequences. Understanding how professional trainers interpret and refine their methods is essential for advancing canine welfare and informing discussions about regulation in an unstandardized profession.

Central to this debate is the ethics and effectiveness of using aversive training methods, including positive punishment and negative reinforcement. Positive punishment introduces an aversive stimulus—such as a shock, leash jerk, or “alpha roll”—to reduce unwanted behaviors, while negative reinforcement removes an unpleasant stimulus, like pressure or noise, when a desired behavior occurs. These techniques are often combined, as with prong collars that apply pressure when a dog pulls and release it when the dog stops. Trainers using these methods have been described as traditional, aversive, or punishment-based. In contrast, positive reinforcement methods, which gained prominence in the 1980s and 1990s, use rewards like treats, toys, or praise to encourage desirable behaviors. Some trainers now adopt the label “balanced” to reflect the use of all four operant conditioning quadrants: positive reinforcement, negative reinforcement, positive punishment, and negative punishment (13, 14).

Positive reinforcement trainers—also called force-free, reward-based, or humane trainers—avoid intentionally using positive punishment or negative reinforcement. Many follow the Least Intrusive, Minimally Aversive (LIMA) guidelines or the Humane Hierarchy (15–17), which begin by assessing a dog’s well-being and adjusting environmental factors that may contribute to problem behaviors. Training primarily emphasizes positive reinforcement and incorporates differential reinforcement strategies, such as rewarding the absence of unwanted behaviors or encouraging incompatible behaviors (e.g., teaching a sit to replace jumping). If problematic behaviors persist, some may apply negative punishment, such as temporarily withholding play, treats, or attention. Reflecting this commitment to humane practices, some organizations grounded in positive reinforcement, such as the Pet Professional Guild (18), have articulated their own position statements and non-negotiable stances against the use of force.

The professional dog training landscape has become increasingly characterized by a politicization of method—that is, the infusion of technical training choices with moral meaning, professional identity, and ideological affiliation (19–21). Debating dog training methods extends well beyond industry circles, unfolding across social media platforms where high-profile trainers command large followings. For instance, proponents of positive reinforcement such as Zak George (22) and balanced trainers like Cesar Millan (23) and The Dog Daddy (24) have each attracted 3 to 4 million YouTube subscribers, with videos that elicit thousands of comments debating the ethics and efficacy of their approaches. Despite the intensity of public debate, empirical evidence remains the most reliable means of evaluating training efficacy and welfare impact. A growing body of behavioral research provides critical insight into how different methods affect canine outcomes—offering a scientific counterpoint to anecdotal and ideologically charged discourse.

### 1.1 Evidence base and limitations

Empirical research underscores the importance of training methods for canine welfare. Studies directly comparing positive reinforcement with methods incorporating positive punishment or negative reinforcement have examined outcomes such as dog behavioral problems, task learning, and physiological stress responses. With the notable exception of one experimental study by Johnson and Wynne (25), the body of evidence consistently shows that positive reinforcement is associated with fewer behavioral issues, including aggression, reduced distress and fear, and effectiveness comparable to or exceeding that of aversive techniques (26–38).^1^ Accordingly, the American Veterinary Society of Animal Behavior (39) recommends that canine training rely exclusively on reward-based methods.

At the same time, much of this literature relies on correlational designs and convenience samples, which restrict causal inference. Additional methodological concerns have also been raised, including a lack of specificity in behavioral definitions, expectancy bias in observational studies, and limited generalizability of the study samples (40, 41). Two out of the three existing quasi-experimental studies (30, 31) exhibited limitations such as lack of procedural control, no pre-post measure analysis, and potentially inadequate e-collar intensity, as noted by Sargisson and McLean (42).

Randomized controlled trials (RCTs) provide the strongest evidence of causality. The only published RCT to date is Johnson and Wynne’s (25) study comparing e-collar training to food reward for preventing lure chasing. While only dogs with activated e-collars ceased the behavior within 5 days, they were also the only ones to yelp during training. Unfortunately, the conclusions that can be drawn from this study are limited by the small sample size, the confound of providing an extra training session to the e-collar group, and the lack of validation for the food reinforcer’s motivational salience when compared to other rewards such as toys.

Cumulatively, the evidence comparing training approaches reveals both strengths and gaps: a broad and consistent association between aversive methods and welfare risks, but methodological imperfections that complicate causal claims. These limitations highlight the need to understand why trainers diverge in their interpretation of evidence and why polarization persists within the profession. Cognitive tendencies such as confirmation bias—the tendency to favor evidence that supports preexisting beliefs while discounting contradictory findings—further exacerbate these divisions (43). At the same time, the absence of standardized oversight or regulatory frameworks may perpetuate these challenges, allowing conflicting interpretations of evidence to translate directly into divergent training practices.

### 1.2 An unregulated profession

The dog training profession remains largely unregulated in most countries, including the United States, leading to substantial variation in trainer qualifications and practices (13, 44, 45). In the absence of standardized oversight, dog owners bear primary responsibility for identifying qualified professionals, which may introduce risks to canine welfare (44). A recent survey of more than 700 Canadian trainers illustrates these challenges. Although most reported using reward-based methods, their educational qualifications varied greatly, and nearly two-thirds (62%) expressed support for some form of regulation (44). In New Zealand, similar concerns have fueled debate over national accreditation frameworks aimed at safeguarding animal welfare and strengthening public confidence in animal training and behavioral modification professions (45). The cases of New Zealand and Canada underscore the importance of examining professional trainers’ perspectives in a largely unregulated industry that is governed only by voluntary credentialing bodies.

### 1.3 Research on dog trainers

Despite growing attention to the ethics and effectiveness of dog training methods, few studies have empirically examined the perspectives and practices of professional trainers. Existing literature largely comprises historical and cultural analyses foregrounding prominent figures in the field (20, 21, 46, 47). Additional research includes an ethnographic comparison of obedience- and reward-based trainers (19) and content analyses of training manuals evaluating the accurate use of scientific terminology (48, 49). In a more comprehensive study of 100 highly rated dog trainers’ online profiles, Johnson and Wynne (13) found that 55% incorporated aversive methods, and two-thirds did not list educational accreditation.

Addressing this gap, we conducted in-depth, semi-structured interviews with 35 trainers affiliated with independent certification bodies. This subset was selected for two primary reasons: first, to explore how trainers committed to professional development diverge in their philosophies and practices; and second, to leverage directories to construct a structured sampling frame, thereby minimizing the bias typically associated with open, self-selected recruitment. This approach facilitated the exploration of emergent themes with a depth and flexibility often absent from purely quantitative research (50, 51).

Participants were identified via a pre-screening survey as current or former users of aversive techniques. Using thematic comparative analyses complemented by inferential quantitative analyses, we explored the factors influencing trainers’ motivations, experiences, and beliefs regarding training method choices and regulation. Particular attention was given to understanding how and why trainers adopt, adapt, or reject specific methods. Guided by these aims, the study addresses the following research questions among trainers sampled from certification-affiliated directories:

1. What results do trainers aim to achieve with their training?
2. What public misconceptions about dog training present challenges for trainers?
3. How important is the emotional well-being of dogs during training sessions?
4. What are their views on the effectiveness of aversive and non-aversive training methods?
5. When and why do trainers use aversive methods?
6. Are there training methods, techniques, or equipment that trainers believe should not be used?
7. What are trainers’ thoughts on the implementation of industry regulation?
8. To what extent are professional development interests, certification status, and gender associated with differences in dog training approaches?
9. What factors contribute to the retention or modification of training methods?
10. Are trainers receptive to changing their methods and techniques?

To enhance the readability of this manuscript, we refer to trainers who incorporate positive punishment or negative reinforcement as *mixed methods trainers*. In contrast, those who intentionally avoid these techniques are termed *positive reinforcement trainers*. The Supplementary material: Survey provides specific definitions and examples of these terms.

## 2 Materials and methods

This study was approved by the Office of Research Compliance and Integrity at Grand Valley State University and was classified as Exempt from full review by the Institutional Review Board on November 13, 2023 (24-093-H).

### 2.1 Procedure

This study was primarily qualitative, with quantitative pre-screen survey data used to complement and contextualize interview findings and to address specific research questions.

#### 2.1.1 Sampling

Three online professional dog trainer directories—the International Association of Canine Professionals (IACP), the International Association of Animal Behavior Consultants (IAABC), and the Certification Council for Professional Dog Trainers (CCPDT)—were used as the sampling frame. The CCPDT serves as a certifying body, while the IACP and IAABC are professional organizations offering optional certifications. The IACP (74) does not endorse a specific training methodology, whereas both the IAABC (52) and CCPDT (15) advocate for the Least Intrusive, Minimally Aversive (LIMA) approach. At the time of data collection, the directories collectively listed approximately 4,509 members with U. S. state affiliations.

Sampling was stratified to reflect the distribution of trainers across the four U. S. Census Bureau regions: West (28%), Midwest (20%), Northeast (23%), and South (29%). To account for potential non-response due to ineligibility, time constraints, disinterest, or outdated contact information, four times the target sample size of 40 trainers were invited to participate.

#### 2.1.2 Pre-screen survey and quantitative analysis

Following selection through stratified random sampling, trainers provided informed consent, were assured of confidentiality, and then completed a pre-screen survey (see Supplementary material) to determine study eligibility. Criteria for participation included: (1) current or prior use of positive punishment or negative reinforcement in dog training; (2) a minimum of 2 years of professional dog training experience; (3) age 18 or older; and (4) residency within the United States at the time of the interview.

Participants reported demographic information, including gender, age, years of professional experience, and average weekly hours devoted to training-related activities (including direct training, teaching, client counseling, and related tasks). They also rated the perceived effectiveness of five training methods—classical conditioning, positive reinforcement, positive punishment, negative reinforcement, and negative punishment—on a 0–4 Likert scale ranging from “Not at all effective” to “Extremely effective.” A “Do not Know” option was available but unused. Participants also reported their history of use for each method: “Never intentionally used,” “Currently intentionally use,” or “Previously used but discontinued,” which assigned participants to one of two interview protocols: trainers who currently use aversive methods and trainers who had transitioned away from them.

Quantitative data were analyzed using JAMOVI (53). These data were used to describe participant characteristics and to address Research Question 4 (perceived effectiveness of training methods) and Research Question 8 (associations between professional characteristics and training orientations). Descriptive characteristics of the final interview sample are presented in Section 2.3.

### 2.2 Qualitative data collection and analysis

#### 2.2.1 Interview procedures

Between January 15 and February 20, 2023, 35 semi-structured interviews were conducted via Zoom or telephone, according to participant preference. Interviews ranged from 45 to 135 min (median = 60 min) and were recorded and transcribed using Otter.ai (54). In exchange for participation, trainers received a $10 gift card or an equivalent donation to the ASPCA.

To mitigate social desirability bias, several strategies were employed. Participants were reminded of their informed consent at the start of the interview, which was conducted as audio-only (unless video was requested) to reduce visual cues. Rapport was established through initial questions about professional background and motivations, followed by open-ended, non-leading questions designed to elicit trainers’ perspectives and experiences. The interviewer (first author) maintained an affirming, genuinely interested demeanor, and many trainers commented on the objectivity and breadth of the interview questions.

The study employed an emergent interview design to allow flexible exploration of trainers’ perspectives. While a predetermined set of questions ensured consistency across participants, the interview protocol was iteratively refined as unanticipated topics arose. For example, although industry regulation was not explicitly included in the initial guide, this topic was incorporated after early participants raised concerns regarding oversight, professional responsibility, and the appropriate use of training tools. This design allowed critical dimensions of professional practice to surface organically within participants’ narratives rather than being strictly predetermined by the researchers.

#### 2.2.2 Qualitative analysis and thematic development

Qualitative data were analyzed using Reflexive Thematic Analysis [RTA; (55, 56)], informed by abductive logic. This approach supported iterative and recursive movement between the study’s research questions—which served as orienting and sensitizing concepts—and inductive openness to patterns of meaning emerging from the data. While the study was guided by 10 research questions, these did not function as a deductive coding framework, and no predefined codebook was used. Transcripts were imported into MAXQDA 24 Analytics Pro (57) for analysis. The analytic process unfolded across three overlapping and iterative phases:

##### 2.2.2.1 Familiarization and preliminary coding

Initial familiarization began during data collection to support reflexivity and responsiveness to emergent topics. This interim analytic engagement identified issues not explicitly anticipated in the interview guide—such as trainers’ interpretations of scientific research and perceptions of industry regulation—as salient to how participants articulated their professional roles and expertise.

##### 2.2.2.2 Systematic coding and refinement

Following completion of all interviews, the lead author conducted systematic coding of the full dataset. Because participants frequently addressed multiple research questions within a single narrative, interviews were coded holistically across the dataset rather than question-by-question. To enhance analytic rigor, manual coding was supported by MAXQDA’s AI Assist tools, which generated suggested codes for analytic comparison and refinement, without replacing interpretive judgment.

##### 2.2.2.3 Thematic construction

To transition from initial coding to thematic construction, MAXQDA’s synthesis features were used to review coded segments across the full dataset. By generating cross-document summaries of related codes, these tools supported comparison of how participants articulated similar concepts. Interpretive analysis of these patterns informed the construction and refinement of overarching themes, which often bridged multiple research questions. For example, while Research Question 8 focused on professional development, the resulting theme *Epistemic Authority* captured broader tensions in how trainers evaluate and privilege scientific knowledge. Similarly, descriptive codes related to dogs’ emotional states (e.g., “lip licking,” “tail tucking”) were synthesized into the subtheme *Assessment and Management of Distress* under the broader theme *Prioritizing Canine Emotional Well-Being*. For clarity of presentation, themes were subsequently organized under the research questions they most directly informed. To enhance analytic transparency, a thematic map (Table 1) illustrates relationships among the study’s research questions, themes, and subthemes.

**Table 1:** Alignment of research questions with themes and subthemes.

Research questions	Themes and subthemes (italicized)
RQ1. What results do trainers aim to achieve with their training?	Fostering a harmonious human-canine relationship
RQ2. What public misconceptions about dog training present challenges for trainers?	Unrealistic expectations of training and development Misreading canine behavior and needs *Breed and lifestyle mismatches* *Assumptions of universal sociability* Confusion about training philosophies and professional standards
RQ3. How important is the emotional well-being of dogs during training sessions?	Prioritizing canine emotional well-being *Assessment and management of distress* *Divergent views on stress as a motivator*
RQ4. What are their views on the effectiveness of aversive and non-aversive training methods?	Perceived effectiveness of training methods
RQ5. When and why are aversive methods used?	Aggression and safety risks Variable application: Efficiency and communication Supporting freedom and safety Ethical concerns and alternatives
RQ6. Are there training methods, techniques, or equipment that trainers believe should not be used?	Skill and application versus the tool itself Differentiating among tools Universal rejection of dominance-based methods
RQ7. What are trainers’ thoughts on industry regulation?	Support for regulatory oversight and professionalization *Preference for education and competency-* *based standards* *Concerns over tool bans and unintended* *consequences* The politicization of professional discourse
RQ8. To what extent are professional development interests, certification status, and gender associated with differences in dog training approaches?	Professional pathways and knowledge foundations in dog training *Commitment to professional development* *Divergent sources of expertise* *Critiques of research methodology* *Certification and gender correlates of training* *approach*
RQ9. What factors contribute to the retention or modification of training methods?	The transition away from aversives: A philosophical conversion *Ethical reflection and empathy* *Exposure to positive reinforcement* *approaches* Transition processes among mixed methods trainers *Increased emphasis on positive reinforcement* *Selective incorporation of aversives* *Pragmatic refinement*
RQ10. Are trainers receptive to changing their methods and techniques?	Adaptation as a professional imperative *Commitment to continuous refinement* *Resistance to methodological change*

Themes and subthemes were developed through reflexive thematic analysis. Themes represent interpretive patterns of meaning that often span multiple research questions, while subthemes capture more specific dimensions. For clarity of presentation, themes are organized under the research question(s) they most directly inform, although analytic development was conducted holistically rather than question-by-question.

#### 2.2.3 Reflexivity and analytic sufficiency

Reflexivity was actively practiced throughout the analytic process. The lead author, a community psychologist and evaluator with professional experience in animal welfare research, and the second author, a professor who studies social cognition, engaged in regular discussions to acknowledge and mitigate potential interpretive bias. Neither author has professional experience as a dog trainer or as a dog owner beyond childhood. This outsider analytic perspective supported sustained attention to participants’ domain-specific expertise and minimized assumption-driven interpretation.

Participant recruitment continued until interviews no longer yielded substantively new insights or conceptually distinct perspectives relevant to the study aims. In line with reflexive thematic analysis, this point in recruitment was understood as analytic sufficiency (56)—the point at which the data provided a rich, complex, and multifaceted account of the phenomena—rather than the mere repetition of identical statements. This judgment was informed by interviewer reflexivity and the recurrence of similar rationales, justifications, and professional narratives across interviews, rather than by formal code counts or predetermined sample thresholds.

### 2.3 Participants

The final sample was derived from the following directories: CCPDT (40%), IAABC (14%), and IACP (46%). Table 2 presents their overall demographics, along with those of each trainer subgroup. Data suggest that our sampling procedure successfully yielded a sample that closely resembled the regional distribution of the directories. Two-thirds of participants identified as women, a finding similar to Zippia’s^2^ (58) estimate of 61% representation within the U. S. dog training profession. Additionally, participants represented a diverse age range (*M* = 47; range = 25–67). Seventy-four percent of trainers reported working 20 or more hours per week, and 80% reported five to 25 years of professional experience, fulfilling our goal of engaging active professionals.

**Table 2:** Demographic and professional characteristics of dog trainers, overall and by training approach.

	Total trainers	Mixed methods trainers	Positive reinforcement trainers
**Age**	(*N* = 35)	(*n* = 18)	(*n* = 17)
Mean	47	46	48
Median	50	50	50
Range	25–67	25–63	25–67
**Gender identity**	*N* (%)	*n* (%)	*n* (%)
Women	23 (66)	11 (61)	12 (71)
Men	12 (34)	7 (39)	5 (29)
**Region of residence**	*N* (%)	*n* (%)	*n* (%)
West	11 (31)	6 (33)	5 (29)
Midwest	6 (17)	6 (33)	0 (0)
Northeast	7 (20)	2 (11)	5 (29)
South	11 (31)	4 (22)	7 (41)
**Weekly training hours**	*N* (%)	*n* (%)	*n* (%)
< 20	9 (26)	2 (11)	7 (41)
≥ 20	26 (74)	16 (89)	10 (59)
**Years in occupation**	*N* (%)	*n* (%)	*n* (%)
2–< 5	4 (11)	2 (11)	2 (12)
5–<15	16 (46)	7 (39)	9 (53)
15–<25	12 (34)	6 (33)	6 (35)
≥ 25	3 (9)	3 (17)	0 (0)
**Training**	*N* (%)	*n* (%)	*n* (%)
Behavioral	31 (89)	17 (94)	14 (82)
Obedience	16 (46)	9 (50)	7 (41)
Service and assistance	6 (17)	4 (22)	2 (12)
Track and scent	2 (6)	1 (6)	1 (6)
Protection and guard	2 (6)	2 (11)	0 (0)
Agility	1 (3)	0 (0)	1 (6)
Performance	1 (3)	0 (0)	1 (6)
Conformation	1 (3)	0 (0)	1 (6)
Therapy	1 (3)	1 (6)	0 (0)
Competitive obedience/rally	1 (3)	1 (6)	0 (0)

Eighteen trainers currently used aversive methods, while 17 did not. Among the latter group, 14 had previously used aversive methods but had since discontinued their use, and three reported never intentionally employing such techniques. These three participants were retained because their survey responses indicated inadvertent use of positive punishment or negative reinforcement; during interviews, they clarified that these instances resulted from trainer error or unanticipated dog responses. Their inclusion provided additional contextual insight into trainer perspectives.

While we captured a variety of trainer specialties, participants commonly indicated that they provided services to decrease unwanted behavior such as reactivity, aggression, or anxiety (89%), increase obedience (46%), and train service animals (17%). Regarding group differences, 94% of mixed methods trainers mentioned providing behavioral training compared to 82% of positive reinforcement trainers. In addition to their specialized dog training work, some interviewees also provided professional development to other trainers through individual or group instruction.

## 3 Results

### 3.1 What results do trainers aim to achieve with their training?

#### 3.1.1 Fostering a harmonious human-canine relationship

Both mixed methods and positive reinforcement trainers shared the goal of strengthening the dog–owner relationship and fostering a more “harmonious and fulfilling life” for both. There was widespread agreement that owner engagement and tailoring training to the needs of each dog–owner pair were essential to success. As one mixed methods trainer explained:

*The overall goal is for the dogs to be happy, and the owners to be happy. What that looks like is different for each dog and each owner... getting the dog and the client on the same page with clear and established communication, clear boundaries, clear structure… just building that relationship between the two of them is what I strive for.*

In pursuit of this outcome, trainers emphasized that their role extended beyond working with dogs to educating owners as well. As a positive reinforcement trainer described:

*My job is to take my understanding of learning theory and canine ecology and help you understand how the environments that we’re creating and our behaviors as humans are impacting what your dog is doing and how we can shape that if there is a change you want to achieve.*

While most participants encouraged owners’ hands-on involvement throughout the process, some mixed methods trainers also offered board-and-train programs, in which owner participation was more limited during the initial stages.

Although trainers across orientations shared a unified vision of fostering mutual understanding and a harmonious bond between dogs and owners, they emphasized that realizing these outcomes is often complicated by owners’ misconceptions about training and canine behavior. Many described their work as not only shaping dogs’ behavior but also recalibrating human expectations and correcting misinformation that undermines progress. The next section explores the most common public misconceptions and misunderstandings that trainers identified as barriers to effective practice and canine welfare.

### 3.2 What public misconceptions about dog training present challenges for trainers?

#### 3.2.1 Unrealistic expectations of training and development

A central challenge reported by both groups was owners’ unrealistic expectations about the speed of behavioral change. Trainers described clients seeking quick fixes rather than recognizing training as a gradual process requiring consistency, patience, and long-term commitment. As one mixed methods trainer observed:

*You have a TV show and the whole behavioral problem seems to be resolved in 30 minutes… It’s not how it really works.*

Trainers emphasized that effective training begins early in a dog’s life and that missed socialization opportunities can have lasting effects. One positive reinforcement trainer explained that many owners misinterpret the concept of socialization, believing it simply means playtime with other dogs:

*People think socialization means taking their dog to the park to run around and play with other dogs—but that’s not what real socialization is. Socialization is exposing the dog to the world: different sounds, the trash truck, the UPS van, the bus, the train, loud noises—all of it. If that puppy isn’t exposed to the big world early on, fear behaviors develop, and those can be really difficult to fix later. Another big issue is that people wait too long to seek help. They only call a trainer once something has gone wrong.*

A mixed methods trainer highlighted the tendency for owners to focus prematurely on obedience skills rather than foundational confidence-building:

*The biggest mistake dog owners make is doing too much, too fast—and not letting dogs just be puppies. I think most people, myself included, have been guilty of that. You get an eight-week-old puppy and want to teach them to sit right away. But that’s not where the focus should be. There’s a lot more foundational work to do first—things that build a stronger, more confident dog. If you’re worried about whether they can sit, lie down, or stand by sixteen weeks, you’re missing what really matters at that stage.*

Trainers collectively emphasized that early exposure, realistic expectations, and patience are central to developing confident, well-adjusted dogs. They expressed frustration that many clients delay training until behavioral problems emerge, rather than seeking guidance proactively during critical developmental periods. Beyond issues of timing and expectation, trainers emphasized that much of their work involved teaching owners the fundamental language of their canine companions.

#### 3.2.2 Misreading canine behavior and needs

Trainers repeatedly noted that many owners struggled to interpret canine communication and behavior without guidance. Anthropomorphism often led clients to misread dogs’ signals, instincts, and needs. For example, one trainer explained that a dog’s “guilty look” is not evidence of wrongdoing but rather a stress response. Both groups described educating owners in canine body language, the importance of daily structure, and the value of consistent communication as central to building a healthy human–dog bond. Several likened this process to parenting—requiring patience, empathy, and understanding rather than expecting automatic obedience.

##### 3.2.2.1 Breed and lifestyle mismatches

Interviewees emphasized the importance of prospective owners understanding breed-specific traits and needs before acquiring a dog. Mismatches—such as pairing a high-drive dog with a sedentary lifestyle—were seen as common sources of frustration and behavioral issues. This challenge directly relates to the broader issue of bridging the gap between owners’ desired outcomes and their dogs’ actual needs. As one mixed methods trainer articulated:

*I don’t know how to take a drive dog and just make it flattened out on a couch. I would have to be extremely cruel to the dog, and I just won’t. I explain to them, ‘Hey, your dog needs a lot more than what you think it needs,’ and try to find some middle ground. Sometimes they’re nervous—they’ve heard rumors like, ‘I shouldn’t play tug of war with my dog, it’ll make them aggressive.’ But if they’ll give me a week or two and start working on tug my way, they usually see that not only is he not getting more aggressive, but he’s actually behaving better in the house.*

##### 3.2.2.2 Assumptions of universal sociability

Another widespread misconception was the assumption that all dogs are inherently social and enjoy human interaction. Trainers stressed that, much like humans, dogs exhibit a wide range of social preferences, and forcing unwanted interactions can lead to stress or behavioral issues.

Together, these misconceptions reveal that trainers view their work as translating canine communication for owners—bridging the gap between human expectations and dogs’ actual behavioral and emotional needs.

##### 3.2.2.3 Confusion about training philosophies and professional standards

Some positive reinforcement trainers noted the misconception that using rewards reinforces undesirable behavior. They emphasized that redirecting or distracting dogs with treats does not reinforce unwanted reactions but instead interrupts and prevents the rehearsal of problematic behaviors. As one interviewee explained:

*If I ask my dog to sit and he chooses to sit, I reward him—that’s an operant response. But most of the time, dogs aren’t thinking before they act; they just act. Redirecting them and distracting them are good things to do, because if that prevents them from reacting further, they’re not practicing that behavior, so they’ll do it less. That’s still kind of counterintuitive to a lot of my clients, but once they see it in action, it opens up a whole new world for people.*

More commonly, trainers from both groups described widespread public confusion stemming from the diversity of training philosophies within the industry. They agreed that the lack of standardized education and regulation makes it difficult for owners to discern which trainers employ ethical and effective methods.

##### 3.2.2.4 Integrative summary

In sum, these accounts underscore that trainers must contend not only with clients’ misunderstandings about dogs but also with public uncertainty about the profession itself—shaped by the absence of consistent regulation and standards. Trainers saw their role as both educators and translators, bridging the divide between scientific knowledge, canine communication, and human expectation to promote more humane and effective relationships between people and their dogs.

### 3.3 How important is the emotional well-being of dogs during training sessions?

#### 3.3.1 Prioritizing canine emotional well-being

Both groups underscored that emotional well-being was central to ethical and effective training. Trainers consistently described confidence and trust as preconditions for progress, positioning welfare not as peripheral but integral to training goals. As one mixed methods trainer explained:

*The emotional state of the dog is 100%, like, the most important thing that I'm looking at, and thinking about when I'm interacting with dogs and training them and teaching them to be confident and overcome their problem behavior.*

A positive reinforcement trainer echoed this view:

*Any animal or person or whatever that's under distress is not in an alert frame of mind.*

Trainers described two interrelated dimensions of emotional well-being: first, how they recognized and managed canine distress during training, and second, how they conceptualized the role of stress in promoting or hindering learning.

#### 3.3.1.1 Assessment and management of distress

Trainers described the ability to read canine body language as a core professional skill. Frequently cited indicators of distress included avoidance behaviors, lip licking, yawning, tail tucking, stiffening, freezing, cowering, postural shifts, disengagement, and reluctance to participate. When such signs appeared, trainers reported adjusting their approach by incorporating breaks, offering enrichment, reducing stimulation, or ending the session.

Group differences emerged in emphasis. Positive reinforcement trainers viewed avoiding aversive methods as a precursor to safeguarding well-being, while mixed methods trainers were nearly unanimous in stating that aversives should never be used when a dog showed distress. As one noted:

*If we’re over threshold, the dog has officially lost its mind, you’re probably not going to see me punish that dog. I’ll probably just abort—the goal is to not get there. If we did, we messed up, and that dog is no longer in a state of learning.*

#### 3.3.1.2 Divergent views on stress as a motivator

Despite this shared commitment to dog welfare, trainers diverged in how they interpreted stress in training. Positive reinforcement trainers characterized stress as detrimental and to be minimized. By contrast, some mixed methods trainers argued that controlled exposure to stress—when paired with guidance and opportunities for success—could help dogs build resilience, acquire coping skills, and adapt more comfortably to life with humans. As one participant explained:

*But you know, as humans, sometimes life is uncomfortable. And sometimes we have to do hard things in order to see the benefit on the other side. Humans go through addiction rehab… nobody likes going to the gym, but if you want to be healthier, you have to go through it…I always look at the end game because sometimes we have to put these dogs through a little bit of discomfort and a little bit of stress in order to get them to see that they don't need to be that stressed all the time. And I’m going to help you through that.*

#### 3.3.2 Integrative summary

In sum, trainers across orientations agreed that safeguarding dogs’ emotional well-being was central to effective practice, but diverged in how they interpreted the role of stress: Positive reinforcement trainers emphasized minimizing distress as fundamental to welfare, whereas mixed methods trainers more commonly viewed stress as context-dependent and, at times, a constructive element of training.

### 3.4 What are their views on the effectiveness of aversive and non-aversive training methods?

#### 3.4.1 Perceived effectiveness of training methods

**Figure 1:**
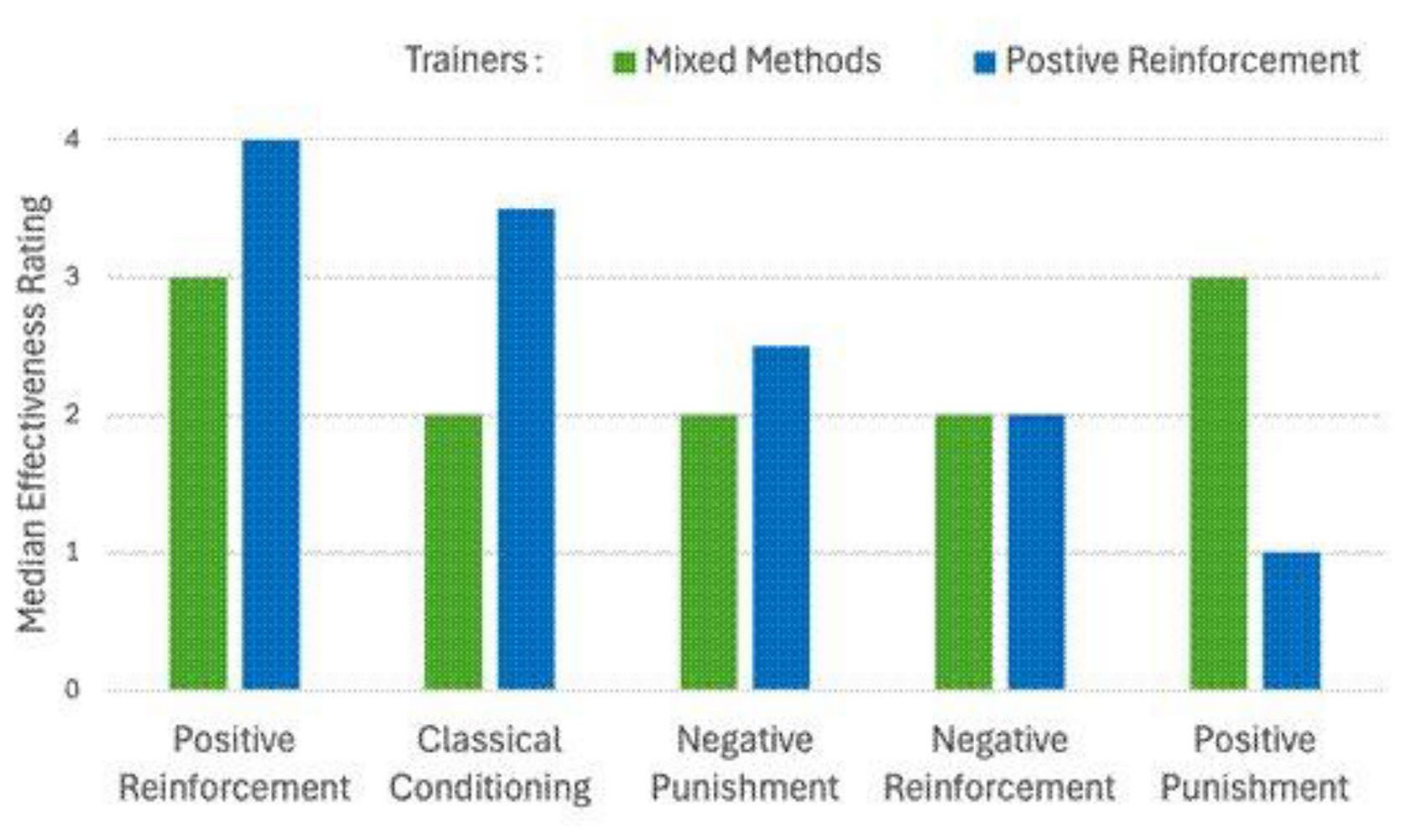
Perceived effectiveness of training technique by trainer type. From left to right, interquartile ranges for mixed methods were 1, 2, 1, 1, 1, respectively. Those for positive reinforcement methods were 1, 1, 1, 0.75, 2, respectively.

Independent samples *t*-tests revealed that both trainer groups rated positive reinforcement as either the most effective or tied for the most effective among the four operant conditioning methods and classical conditioning (Figure 1). While positive reinforcement trainers reported a slightly higher median effectiveness rating, this difference was not statistically significant. However, compared to mixed methods trainers, they rated classical conditioning significantly more effective and positive punishment significantly less effective (*p* < 0.05). Notably, positive reinforcement trainers also showed considerable variability in their ratings of positive punishment, suggesting differing views on its effectiveness or ethical acceptability. See Table 3 and Figure 1.

**Table 3:** Independent samples mann–whitney test statistics on perceived training method effectiveness.

Method	Mann–Whitney U Statistic	*p*-value	Effect Size: Rank-Biserial Correlation
Positive reinforcement	105.0	0.078	0.31
Classical conditioning	76.5	0.008**	0.50
Negative punishment	130.5	0.443	0.15
Negative reinforcement	120.5	0.264	0.21
Positive punishment	77.0	0.011*	0.50

*p < 0.05; **p < 0.01.

### 3.5 When and why are aversive methods used?

#### 3.5.1 Aggression and safety risks

Safety was a critical consideration across perspectives. Mixed methods trainers frequently cited aggression, reactivity, and situations involving potential risk to handlers or dogs as contexts where positive reinforcement alone was insufficient. In such scenarios, aversives were viewed as a means of establishing immediate control and preventing escalation.

Positive reinforcement trainers acknowledged that emergencies might warrant temporary use of aversive methods. Approximately one-third stated that interventions such as breaking up a dog fight or preventing a life-threatening incident could justify aversive action. These trainers nevertheless emphasized that such use should remain exceptional and not be normalized as part of training practice.

#### 3.5.2 Variable application: efficiency and communication

Mixed method trainers held varied perspectives on the application of aversives. While some regarded these methods as a last resort, implementing them only when a dog’s behavior posed an immediate risk to itself or others, others integrated them more routinely. In this sense, aversives were considered efficient aids for clear communication and speed, particularly in contexts such as leash walking, bite prevention, and off-leash recall training. One trainer articulated this perspective regarding efficiency, noting:

*I won’t say it [positive reinforcement] doesn’t work, I just found that it’s more realistic to use some correction as little as possible to speed things up, and just to make it clear for the dog.*

#### 3.5.3 Supporting freedom and safety

Aversive methods were also frequently highlighted by mixed methods trainers for their role in supporting freedom and safety during off-leash activities. As one trainer explained:

*No amount of food reward can compete with the innate desire to chase prey, and in such moments [a dog with a high prey drive chasing a squirrel], a negative reinforcement routine using an e-collar can make a command more reliable.*

This was linked to a broader philosophical argument about expanding dogs’ freedom to aid emotional stability:

*If you think back just 60 years ago, people opened the door in the morning and let their dogs out. They ran around, played with other dogs and children, and even went to market off-leash. Dogs were more integrated into society, and you didn’t see the same aggression issues we see today.*

#### 3.5.4 Ethical concerns and alternatives

By contrast, positive reinforcement trainers rejected the notion of efficiency and freedom as valid rationales for aversive method use. They argued that the perceived expedience of tools like e-collars was outweighed by the risk of harm and the availability of safer management strategies. They consistently cited risks of fear, anxiety, and negative health or behavioral outcomes, questioning both the moral acceptability and long-term effectiveness of aversive methods. Even when acknowledging that size or resilience might influence a dog’s immediate response to aversive methods, they rejected trainer preference or breed as justification for usage. Instead, they pointed to management strategies such as long-line leashes as humane alternatives. One trainer illustrated this perspective:

*The goal, I guess, is great, but I don’t really think that should be the goal. If your dog runs, just put them on a 50-foot leash—it’s really not that big a deal. I’ve done it with a lot of dogs. They don’t know they’re on a leash, they’re still able to explore as much as they can. You have that safety.*

#### 3.5.5 Integrative summary

Together, these findings reveal a broader philosophical divide. For mixed methods trainers, aversives were pragmatic instruments that, when skillfully employed, could enhance communication, expedite training, and even expand dogs’ freedom. For positive reinforcement trainers, aversive use represented an ethical compromise tolerated only under extreme circumstances, with management strategies preferred to correction. While both groups recognized that safety risks could override training ideals, their broader orientations diverged: mixed methods trainers emphasized efficiency and balance, whereas positive reinforcement trainers prioritized minimizing exposure to aversive stimuli.

### 3.6 Are there training methods, techniques, or equipment that trainers believe should not be used?

#### 3.6.1 Skill and application versus the tool itself

Among mixed methods trainers, a recurring theme was that the acceptability of training tools depended less on the equipment itself and more on the trainer’s knowledge, timing, and application. Participants emphasized that misuse by unskilled individuals, rather than the inherent properties of a tool, was the primary source of harm. From this perspective, responsible use was inseparable from professional expertise, underscoring the need for education and careful application in order to minimize risk. As one trainer stated:

*I won’t say no to any tool, I think any trainer worth their salt needs to have a pretty diverse toolbox. And more important than that, they need to know how these tools are supposed to be used. Because I see more people misusing things like prong collars than anything else.*

Positive reinforcement trainers tended to disagree with this perspective, viewing the problem as intrinsic to the tools rather than rooted in trainer competence. They opposed the use of aversive equipment such as electronic collars, prong collars, and choke chains outright. One positive reinforcement trainer reflected on the types of equipment they most preferred not to see in practice:

*The dog will sort of choke himself out and stop when he can't breathe anymore. I don’t like to see collars used in that way. I have concerns about e-collars, whether we’re calling them e-collars, stim collars, you know, shock collars. I am seeing some clients who use muzzles that hold the dog’s mouth shut. I do not like to see those. I feel like those are physiologically pretty dangerous for the dog and really aversive.*

#### 3.6.2 Differentiating among tools

Despite their emphasis on trainer skill, mixed methods trainers did not regard all equipment equally. Roughly one-third expressed explicitly negative views of choke chains, citing their potential for injury and limited training value. Slip leads and Martingale collars were consistently described as safer and more effective alternatives. Opinions on e-collars and prong collars were more divided. Some trainers rejected their use entirely due to injuries such as burns, while others conditionally accepted them under circumstances where they could be applied with precision and expertise. This variation reflected ongoing debate within the group about whether certain tools could ever be reliably separated from misuse.

#### 3.6.3 Universal rejection of dominance-based methods

Although perspectives varied on specific collars and electronic devices, there was uniform condemnation of certain practices. Across both groups, alpha rolls, dominance-based physical manipulation, and direct violence (such as hitting) were unequivocally rejected as harmful and unacceptable. These methods were considered beyond the bounds of contemporary professional practice. A mixed methods trainer powerfully encapsulated this rejection, highlighting both the philosophical dismissal of dominance and the moral outrage at abusive actions:

*Alpha rolling, that is the biggest–pardon my language–bullshit that I've ever seen. I don’t see any advantage of using that. Forcing a dog into submission, I don't believe in that…Last week, there was a video that was going viral about a dog trainer who dumped a bucket of water on a German Shepherd in a crate. Yeah, that guy needs to go to jail.*

#### 3.6.4 Integrative summary

While perspectives differed among mixed methods trainers on whether certain equipment could be justified with appropriate skill, there was broad agreement that some practices were unacceptable in professional training.

### 3.7 What are trainers’ thoughts on industry regulation?

#### 3.7.1 Support for regulatory oversight and professionalization

Both mixed methods and positive reinforcement trainers underscored the risks posed by unqualified practitioners and expressed broad support for greater oversight of the dog training industry. They highlighted that the absence of barriers to entry allows anyone to adopt the title of “trainer,” exposing dogs and owners to harmful or outdated practices. Across groups, trainers emphasized that regulation should elevate education, certification, and public awareness—strengthening both professional credibility and animal welfare.

Several participants explicitly supported government-led or third-party oversight, arguing that external regulation could legitimize the field beyond voluntary membership associations. As one positive reinforcement trainer explained,

*I absolutely think there should be regulation—governmental regulation of dog training. Not to dictate methods, but to make sure trainers understand the science behind what they do.*

#### 3.7.1.1 Preference for education and competency-based standards

The most commonly articulated priority across both groups was the need for regulatory standards that emphasize competence and knowledge. Trainers frequently argued that a mandatory, consistent knowledge base—covering operant theory, classical conditioning, and applied behavior analysis—was the best way to ensure humane and effective practice. Many advocated for a testing and licensing system similar to other skilled trades to hold practitioners accountable for malpractice.

Several positive reinforcement trainers referenced existing certification bodies such as the CCPDT, Association for Professional Dog Training, and IAABC, recognizing their value in promoting science-based training while also noting significant limitations. They observed that these organizations lack enforcement mechanisms and that the growing number of credentialing programs can confuse the public and dilute credibility. As one participant stated,

*There’s such an alphabet soup of letters behind our names that it doesn’t mean anything to anyone.*

A few noted concern that current certification pathways may reinforce privilege, as participation often depends on financial means and access to mentorship.

Across groups, participants suggested that public education on learning theory and canine behavior may, in the short-term, be the most practical way to raise standards. As one trainer articulated,

*The best way for regulation to succeed is through public education—helping clients understand what ethical, effective training actually looks like.*

#### 3.7.1.2 Concerns over tool bans and unintended consequences

The most significant philosophical divide centered on whether regulation should restrict or ban specific training tools. Mixed methods trainers were more likely to caution against prohibition, emphasizing that restrictive policies could have unintended negative effects—such as driving harmful practices underground, encouraging misuse of “approved” tools, or contributing to higher euthanasia rates among dogs with severe behavioral issues. One trainer warned,

*I worry about dogs—especially aggressive ones—that are now going to be euthanized because people don’t know how to handle them.*

Another echoed this concern, advocating instead for education and responsible application:

*I don’t think there are any techniques or tools that should flat-out never be used. There’s always that one case where a tool could save a dog’s life. In the hands of an experienced practitioner, I’d rather see more emphasis on how to use these tools safely.*

Positive reinforcement trainers expressed more ambivalence. While some favored banning aversive tools, others questioned whether bans would meaningfully improve welfare without strong educational support or consistent enforcement. As one trainer reflected,

*Whether [aversive tools] should be completely banned—I don’t really have a great answer for that. I see people using them with a very low level of skill, and I just have a lot of concerns, ethically and physiologically, for the dogs on the receiving end.*

#### 3.7.2 The politicization of professional discourse

Beyond the debate over tools, many trainers—particularly those using mixed methods—described a divisive professional climate marked by hostility between training communities. Several recounted being labeled “abusive” or “unethical” online, likening the tone of debate to political polarization. They viewed this conflict as counterproductive to collective progress, arguing that open dialog and respect for contextualized practice were essential to advancing canine welfare. This frustration with the professional climate was clearly articulated by one mixed methods trainer:

*It feels like politics—you get attacked for even talking about methods that aren’t purely positive. I just want more collaboration, not more fighting.*

Another described finding common ground despite methodological differences:

*One of my best friends is purely positive and, as we established, I am not. We can actually have constructive dialogue about training and it's not finger pointing. Her and I constantly talk about how can we bring the camps together. … At the end of the day, it doesn't matter what tools you use, or how you train. As long as you are helping dogs and helping owners, then we're working towards the same goal.*

While some positive reinforcement trainers regarded polarization as a necessary distinction that clarified ethical boundaries within the profession, many also perceived it as divisive and counterproductive to collaboration and shared welfare goals, often attributing differing approaches to gaps in education rather than malice.

#### 3.7.3 Integrative summary

Trainers across methodologies expressed near-universal support for professional regulation to mitigate the risks posed by unqualified practitioners. However, consensus fractured around the form regulation should take. Positive reinforcement trainers generally favored competency-based licensing and, in some cases, bans on aversive tools. Mixed methods trainers, by contrast, emphasized education, professional responsibility, and flexibility to manage complex behavioral cases. Across perspectives, participants called for regulation that promotes collaboration, accountability, and public trust—and highlighted public education as a crucial pathway to improving welfare, reducing misinformation, and strengthening the legitimacy of the profession.

### 3.8 To what extent are professional development interests, certification status, and gender associated with differences in dog training approaches?

#### 3.8.1 Professional pathways and knowledge foundations in dog training

##### 3.8.1.1 Commitment to professional development

Both groups stressed the importance of continued professional development. Trainers reported having engaged in a blend of formal education, informal learning, mentorship, and hands-on experience. Many attended structured certification programs, workshops, and pursued independent study through books, articles, and webinars. Employer-supported continuing education was also noted, highlighting the role of workplace investment in professional growth.

##### 3.8.1.2 Divergent sources of expertise

A key difference emerged in the experts and theoretical lineages each group drew upon. Many positive reinforcement trainers referenced figures widely recognized within the academic field of animal learning and behavior, such as Ken Ramirez, Karen Pryor, Jean Donaldson, and Patricia McConnell. In contrast, mixed methods trainers more often cited figures recognized primarily within the practitioner community, including Michael Ellis, Heather Beck, Jack Jay, Larry Crone, and, to a lesser extent, Cesar Millan—although his methods were sometimes contested. This divergence in foundational expertise also extended to how trainers interpreted scientific literature.

##### 3.8.1.3 Critiques of research methodology

Mixed methods trainers who cited scientific studies often focused on research exploring the long-term impacts of specific aversive techniques, such as using e-collars to reduce predatory chasing, or studies comparing the efficacy of different aversive methods. One participant recounted a six-year study to reduce dogs from chasing Kiwi birds in New Zealand:

*[Electric shocks administered once a year] stopped 1156 dogs from chasing Kiwi birds, an endangered species. Over 850 dogs stopped chasing after initial sessions… In year four was the last group that still chased, a very small number — around 40, I think — and then by year five, nobody chased anything. And all these dogs are perfectly fine.*

When discussing research directly comparing positive reinforcement with mixed methods, mixed methods trainers often provided methodological critiques such as inadequate variable quantification, artificial testing environments, reliance on surveys, and selective data presentation. As one trainer remarked:

*A lot of the information gathered out there about purely positive vs. shock vs. whatever – so much of the studies are so just so skewed and terrible. They’re either done in isolation where they put a dog in the room and electrocute it and see if it can get out or feed it until it pushes the button, or it’s survey-based.*

Another questioned the way stress was operationalized in comparative studies:

*One of the big things is that you should only use two quadrants [positive reinforcement and negative punishment] because the others are bad, but the definition of bad is that there is stress observed and it doesn't quantify the level of stress. I will never see stress as an unproductive emotion. I think it's something that sometimes we need or you know, so then that makes that entire study invalid to me, because their parameters are not something that I consider valuable in the first place.*

These critiques illustrate how some trainers challenged research they viewed as misaligned with their own definitions of welfare and efficacy, reflecting a broader tension between scientific findings and practitioner-based knowledge.

##### 3.8.1.4 Certification and gender correlates of training approach

A chi-square test of association was conducted to examine the relationship between trainers’ certification type (independent body, vocational school, or no formal certification) and their training approach (positive reinforcement vs. mixed methods). Certification data were compiled through online searches and interviews, with the highest credential recorded when multiple certifications were held. Results indicated a statistically significant association between certification type and training approach, *χ^2^* (2, 35) = 15.2, *p* < 0.001, with a strong effect size (Cramér’s *V* = 0.66). All 17 positive reinforcement trainers held credentials from independent certification bodies, whereas mixed methods trainers were most likely to hold certification from a vocational school (44%, *n* = 8), followed by an independent body (39%, *n* = 7), and a minority reported no formal certification (17%, *n* = 3).

A second chi-square test examined the association between gender and training approach. Among the 35 participants, men (*n* = 12) more commonly used mixed methods (58%), whereas women (*n* = 23) more commonly used positive reinforcement (52%). However, this relationship was not statistically significant, *χ^2^* (1, 35) = 0.35, *p* = 0.555.

#### 3.8.2 Integrative summary

Taken together, these findings suggest that professional development and certification intersect with trainers’ methodological orientations in nuanced ways. Positive reinforcement trainers drew more heavily from academic authorities and independent certification bodies, aligning their practices with scientific literature and formal standards. Mixed methods trainers, by contrast, were more likely to rely on practitioner-based authorities and vocational training, and often critiqued research they perceived as disconnected from real-world contexts. While certification type showed a strong association with training approach, gender did not.

### 3.9 What factors contribute to the retention or modification of training methods?

#### 3.9.1 The transition away from aversives: a philosophical conversion

The most common developmental trajectory involved the 14 positive reinforcement trainers who reported previously using positive punishment or negative reinforcement. Their shift was typically marked by growing empathy toward the dogs they trained, ethical reflection, and exposure to alternative learning models that reshaped their understanding of what constitutes effective and humane training.

#### 3.9.1.1 Ethical reflection and empathy

Approximately two-thirds of trainers who transitioned away from aversive methods stated that observing canine distress, fear, or reactivity prompted a reevaluation of their ethical stance. Witnessing behaviors such as shutdown or aggression led many to question whether aversive methods were consistent with their professional values and their core goal of improving canine welfare.

##### 3.9.1.2 Exposure to positive reinforcement approaches

Many trainers initially regarded aversives as standard practice but began to question their use after engaging with reward-based trainers, attending specialized workshops, or learning about modern behavioral science. For many, this exposure represented a pivotal “aha” moment that reshaped their understanding of humane and effective practice:

*I was working with a trainer who was pretty highly rated in the area—very punitive methods. And it just kind of was like, this doesn’t feel right to me... I connected with someone who was force-free who opened up a whole new world for me. I honestly didn’t know that that was possible.*

While a modest majority (58%) of trainers exposed to positive reinforcement approaches described an immediate transition, the remainder reported a more gradual shift as they deepened their understanding of canine psychology and observed more stable behavioral outcomes. Regardless of pace, trainers consistently emphasized that positive reinforcement yielded calmer, more confident, and more responsive dogs—though some noted that it sometimes required greater time and patience.

#### 3.9.2 Transition processes among mixed methods trainers

Mixed methods trainers described three distinct pathways through which their training philosophies and practices changed over time.

##### 3.9.2.1 Increased emphasis on positive reinforcement

The most prevalent pathway (44%) involved trainers who began with correction- or compulsion-based methods and later shifted toward greater reliance on positive reinforcement as they recognized its effectiveness and the potential adverse effects of corrections. Much like the positive reinforcement trainers described earlier, this shift was driven by experience and continuing education, leading to greater emphasis on motivation and relationship-building while reserving aversive tools for specific, limited contexts.

##### 3.9.2.2 Selective incorporation of aversives

A smaller subset (28%) described a movement in the opposite direction—starting from a primarily positive reinforcement framework and later integrating positive punishment or negative reinforcement. They cited perceived benefits for both training outcomes and canine welfare. One trainer reflected on her experience after learning more about various tools:

*Prong collars have been a really wonderful addition to my training tools… I work with people who physically can’t handle their dogs, and using that tool makes them able to stay in their home. As I’ve grown and entered the real world, I feel like, just like with everything in school, you’re taught something and you get out in the real world and realize it’s not always ideal. We’ve got time limits. We’ve got other circumstances.*

##### 3.9.2.3 Pragmatic refinement

The third trajectory (28%) involved ongoing fine-tuning of mixed techniques based on experience. Trainers in this group emphasized improving timing, precision, and communication while maintaining both positive and corrective elements.

#### 3.9.3 Integrative summary

Across both groups, trainers described a common movement toward increased use of positive reinforcement, guided by empathy, ethical reflection, and expanding knowledge of learning theory. Among mixed methods trainers, however, this evolution was more heterogeneous, encompassing varied blends of reinforcement- and correction-based strategies. Across both groups, adaptation was described as an iterative, experience-driven process grounded in effectiveness, professional growth, and a sustained commitment to canine welfare.

### 3.10 Are trainers receptive to changing their methods and techniques?

#### 3.10.1 Adaptation as a professional imperative

Most trainers expressed a strong receptivity to modifying their methods and techniques. The primary catalysts for adjustment included concerns about dog distress, the perceived effectiveness of current training, the owner’s capacity to implement the recommended plan, client expectations, and exposure to new empirical research. Only slight differences in the nature of receptivity emerged between the two trainer groups.

##### 3.10.1.1 Commitment to continuous refinement

The vast majority of trainers described a dedication to ongoing learning and technique refinement. Most mixed methods trainers (83%) explicitly reported this commitment. Their willingness was often framed as an imperative to stay informed and avoid dogmatic thinking, even when they disagreed with particular approaches:

*I want to see what people are doing. I want to be informed. I watch the videos too... I think what happens in the training, unfortunately, we see so much more politicization now than ever before. It's become like, you know, Republicans and Democrats... I think that's what's happening in dog training too. Stealing the narrative.*

They also challenged advocates of force-free or reward-based approaches to provide superior alternatives, indicating that their methodological commitment was conditional on emerging evidence:

*If someone can figure out how to create the same reliability as a prong collar or shock collar over certain situations... sign me up, I'll take the course. I want to learn. But it doesn't exist today.*

Similarly, all positive reinforcement trainers described a willingness to reassess and refine their methods, particularly if current strategies failed to produce desired behavioral changes or caused distress to the dog. As previously mentioned, a small number acknowledged that positive punishment might be considered in rare cases—such as managing proactive aggression or immediate safety concerns—though they emphasized the lack of current empirical support and the need for careful ethical consideration before any deviation.

##### 3.10.1.2 Resistance to methodological change

Despite this broad openness to refinement, a minority of mixed methods trainers (*n* = 3) were less receptive to methodological change, expressing skepticism toward new industry trends. Two suggested that many contemporary approaches merely “reinvent the wheel” rather than offering meaningful advancements.

The third, who aligned with a pack-leadership model, explained that exposure to alternative methods had only reinforced confidence in their original philosophy:

*I’ve found it to be the most effective and the most natural for me…while minor adaptations might be incorporated, the foundational philosophy of my training will remain unchanged.*

#### 3.10.2 Integrative statement

Across both groups, receptivity to methodological change was a defining feature of professional practice, though it varied in degree and framing. All positive reinforcement trainers described openness to adjustment, while most (83%) mixed methods trainers expressed similar commitment to refinement and evidence-based learning. The small subset who resisted change viewed their established philosophies as already optimized, marking the tension between openness to revision and enduring professional conviction. Collectively, these patterns underscore that the motivation to evolve—whether through adoption, calibration, or defense of existing methods—appeared rooted in the majority of trainers’ shared concern for effectiveness and canine welfare.

## 4 Discussion

This study offers one of the first empirical examinations of professional dog trainers’ perspectives across differing methodological orientations, illuminating how ethics, evidence, and professional identity intersect in shaping canine training practices. Drawing on semi-structured interviews and survey data from trainers affiliated with independent certification bodies, the findings reveal both convergence and divergence across groups.

A notable finding was the considerable overlap in perspectives and experiences of positive reinforcement and mixed methods trainers. Both groups articulated a primary objective of improving the dog-owner relationship through individualized training that addresses both canine and human behavior. Furthermore, they emphasized the importance of dogs’ emotional well-being, relied most heavily on positive reinforcement, encountered difficulties with owners’ unrealistic expectations, and shared anxieties regarding unqualified or abusive practitioners. These shared values align with evidence of a cultural shift toward recognizing dogs’ physical and emotional needs beyond instrumental use and the benefits of an authoritative training style (20, 21, 59).

### 4.1 Epistemology, professional pathways, and welfare pluralism

Participants articulated a dynamic philosophical approach to practice, continuously refined through experience, ongoing education, and reflection on each dog’s emotional state. Their applied ethics were evidence-informed yet pragmatic: methods were reported as consistently judged by their perceived effects on canine welfare, behavioral outcomes, and the quality of the human–dog relationship.

Trainers’ moral reasoning reflected a spectrum between deontological and consequentialist (utilitarian) orientations common in applied ethics (60). Positive reinforcement trainers tended to articulate deontological leanings, viewing the intentional use of fear or pain as intrinsically wrong and inconsistent with their duty to respect canine welfare and autonomy. Yet even among this group, ethical reasoning was rarely absolute; many described adapting their methods to situational constraints—such as owner capability, risk level, or the dog’s learning history—suggesting a pragmatic balancing of principle and outcome. Mixed methods trainers tended toward more explicitly utilitarian reasoning, justifying the use of aversive methods under specific conditions where they believed the benefits (e.g., safety, behavioral stability, prevention of relinquishment) outweighed temporary discomfort. These overlapping yet distinct ethical logics demonstrate how trainers who share fundamental welfare goals can reach divergent conclusions about what constitutes “humane” or ethically justified practice, underscoring that ethical disagreement often reflects differing moral reasoning frameworks rather than empirical misunderstanding alone.

These ethical orientations corresponded to distinct epistemic foundations—that is, to what each group regarded as credible knowledge. For positive reinforcement trainers, learning theory and welfare science served as moral and methodological anchors. Mixed methods trainers, by contrast, placed comparable weight on experiential knowledge, grounded in years of field observation and reinforced by perceived real-world outcomes. This divergence reflects a broader tension between academic and practice-based authority characteristic of many applied professions (61).

This epistemic split was also evident in trainers’ developmental pathways. Positive reinforcement trainers almost exclusively referenced academic authorities and experts in learning theory (e.g., Karen Pryor), whereas mixed methods trainers often cited practitioner-mentors and experiential sources (e.g., Michael Ellis). When discussing research, mixed methods trainers critiqued methodological limitations in existing studies, and frequently referenced research that lacked a reward-based condition. Such reflections illustrate how differing sources of authority may shape trainers’ confidence in their methods and inform their interpretations of the evidence.

Certification pathways reinforced these epistemic distinctions. Positive reinforcement trainers sought independent bodies emphasizing formal education in learning theory (e.g., CCPDT, IAABC), contrasting with mixed methods trainers’ more varied routes through vocational programs, apprenticeships, or informal mentorship. This pattern aligns with prior findings (13) and suggests that formal education in learning theory may reduce endorsement of aversive methods (62). Alternatively, trainers may self-select educational pathways that align with their preexisting philosophies—or, more plausibly, epistemic orientation and education interact dynamically to shape both training methods and professional values.

Gender identity was not associated with a specific training approach in this study, suggesting that methodological preferences are more strongly influenced by factors such as certification, experience, and philosophical orientation. In contrast, Johnson and Wynne (13) identified gender as a significant predictor, with women being 2.7 times more likely to hold professional certifications and 2.5 times more likely to employ non-aversive techniques than men. Similar patterns have been reported among dog owners, with men more likely to employ aversive training techniques (27, 63).

Taken together, these findings provide empirical support for perspectival pluralism in animal welfare (64). The necessity for trainers to negotiate between moral conviction and practical necessity—balancing positive welfare outcomes with the immediate avoidance of suffering—demonstrates that the profession operates at the intersection of science, ethics, and lived experience. This interplay among education, experience, and ethical reasoning forms the foundation of methodological evolution and remains a key point of ethical deliberation within the profession.

### 4.2 Philosophical change and adaptive practice

The majority of trainers in both groups described an evolving commitment to positive reinforcement, often motivated by empathy, ethical reflection, and exposure to alternative frameworks. For many positive reinforcement trainers, abandoning aversive methods represented a moral turning point—a transition from behavior control to cooperative teaching. Mixed methods trainers exhibited more heterogeneous trajectories: some also shifted toward reinforcement-based practices, while others selectively incorporated positive punishment or negative reinforcement or refined their existing techniques.

This process of evolution is consistent with the Transtheoretical Model of Behavioral Change (65), which conceptualizes change as a staged progression driven by increasing awareness, self-efficacy, and perceived benefits of adopting new behaviors. While some trainers described gradual, experience-driven change, others recounted more abrupt transitions prompted by moments of moral conflict or observed canine distress. Collectively, these narratives suggest that methodological evolution—whether toward, away from, or within current practices—was guided by ongoing moral and empirical appraisal rather than allegiance to ideology. This finding underscores the role of humility and continued education in ethical decision-making among these types of professional dog trainers.

### 4.3 Regulation, professional legitimacy, and polarization

Regardless of training method, there was near-universal support for stronger professional regulation to mitigate the risks posed by unqualified practitioners, safeguard animal welfare, and enhance the field’s credibility. This finding presents a notable contrast to recent studies in Canada (44) and New Zealand (45), where support for professional regulation or accreditation was more prevalent among trainers who used reward-based methods and held higher qualifications. Consensus was fractured over the *form* regulation should take. Positive reinforcement trainers generally supported licensing and, in some cases, bans on aversive tools, while mixed methods trainers emphasized education, mentorship, and practitioner discretion. Both groups advocated for competency-based standards grounded in behavioral science, yet diverged in whether regulation should prescribe specific methods or allow professional flexibility.

This tension reflects a deeper issue of professional legitimacy—who defines humane practice and on what epistemic grounds. As with other applied professions, trainers operate within plural moral frameworks that yield competing definitions of “best practice.” For some, particularly positive reinforcement trainers, regulation was seen as essential for public trust and animal protection; others, especially mixed methods trainers, feared that top-down governance could marginalize skilled practitioners or overprioritize formal credentialing systems that privilege specific training philosophies. These views reenforce the idea that effective welfare governance be enacted in ways that allow for moral pluralism [cf. (66, 67)] rather than rigid, binary enforcement.

Many participants also viewed public education as integral to effective regulation. Trainers argued that informed owners are less likely to seek coercive methods and more capable of recognizing qualified professionals. Similarly, a recent study indicates that many dog owners overestimate their training knowledge while underestimating the welfare and learning implications of aversive or dominance-based methods (68). This theme positions trainers not merely as service providers but as translators of behavioral science—mediating between research, policy, and everyday practice to improve welfare literacy and consumer decision-making.

Beyond regulatory frameworks, participants described an increasingly polarized professional climate, reflecting a division that mirrors concerns recently documented among Canadian trainers (44). Overall, trainers called for a balanced approach to regulation—one that combines competency-based licensing, public education, and practitioner flexibility that avoids prescribing specific methods.

### 4.4 Implications for professional cohesion and public understanding

The convergence of shared ethical priorities, despite divergence in epistemology and method, highlights two key implications for advancing the profession: fostering internal cohesion and bridging the translational gap between behavioral science and public understanding.

Despite their philosophical divides over tool use and the perceived effectiveness of positive punishment, trainers shared common goals: enhancing owner education, preventing harm, and elevating professional standards. These common aims provide a constructive basis for moving beyond adversarial discourse. Professional associations could increasingly implement perspectival pluralism (66) opportunities through structured cross-engagement initiatives—such as mentorship exchanges, job shadowing, and collaborative workshops. These efforts could promote mutual learning and foster epistemic humility across methodological divides. For instance, mixed methods trainers could observe the effective application of positive reinforcement and classical conditioning in complex cases, while positive reinforcement trainers could gain insight into the decision-making, timing, and welfare assessment accompanying the use of aversive methods.

Trainers’ emphasis on owner education highlights a persistent translational gap between behavioral science and public understanding. High quality education initiatives may enhance welfare literacy, reduce behavior-related relinquishment, and reinforce behavioral science as a cornerstone of humane education (2, 9, 10). For example, trainers from both orientations might collaborate to educate the public about canine behavior, learning theory, breed-specific traits, and the importance of early socialization. Joint efforts to identify and discourage harmful or punitive practices would further clarify ethical boundaries and strengthen professional legitimacy. Such cooperation has the potential to shift discourse away from methodological polarization toward shared welfare goals, thereby fostering professional credibility and public trust.

### 4.5 Study limitations

While this study provides one of the most detailed accounts of professional dog trainers’ perspectives to date, several limitations should be acknowledged. First, although the use of stratified random sampling enhanced rigor relative to online convenience sampling, participation was limited to trainers listed in the IACP, IAABC, and CCPDT directories who were willing to complete interviews. This likely introduced a degree of self-selection bias, skewing the sample toward professionally engaged trainers who value formal education and credentialing—particularly those affiliated with CCPDT, where active certification is required for listing.

Second, to achieve the study’s aim of exploring methodological transitions and the rationale for using various training methods, participants were intentionally identified through a pre-screening survey as current or former users of aversive techniques. While this approach ensured depth of perspective across the methodological spectrum, it resulted in a deliberate underrepresentation of trainers who have exclusively employed reward-based approaches throughout their careers.

Third, although the sample size of 35 participants is suitable for comparative thematic analysis (56), it does not permit statistical generalization to the broader dog training population. The quantitative comparisons by gender, certification type, and methodological orientation should therefore be interpreted as descriptive of this sample rather than as population-level estimates. Consequently, the findings reflect the views of highly engaged practitioners with varied, complex experiences rather than the full diversity of trainers or members of the public.

Finally, as with all self-reported research, the potential for social desirability bias cannot be excluded. Despite efforts to mitigate this through assurances of confidentiality, video-off interviews, and a nonjudgmental interview style, some participants may have provided responses aligned with perceived social or professional expectations. Nonetheless, such biases are not unique to qualitative inquiry and similarly affect survey and experimental research.

Despite these constraints, the study’s stratified sampling design, depth of qualitative inquiry, and integration of comparative analysis provide a strong empirical foundation for future research—particularly regarding the psychological, ethical, and epistemic dimensions of trainers’ professional development and decision-making.

### 4.6 Future research

#### 4.6.1 Investigating perception and efficacy

Future research should examine how trainers’ ethical orientations and epistemic assumptions influence their perceptions of canine distress and training efficacy. Given the aforementioned relationship between aversive methods and behavioral issues such as aggression, fear, distress, and reduced expectancy of positive outcomes, it would be useful to investigate how trainers interpret and respond to signs of distress. Experimental designs could employ video-based paradigms in which trainers evaluate canine behavior and physiological stress indicators while blinded to the training method used, enabling comparison across methodological orientations. Such designs would help determine whether trainers with differing methodological orientations interpret distress signals differently.

Longitudinal and experimental methods should also be used to address the efficiency hypothesis—specifically, testing the assumption that reward-based approaches inherently require longer durations to achieve critical behavioral change (e.g., stopping predatory chasing).

#### 4.6.2 Developing ethical frameworks

Future research grounded in psychological theory should examine the moral foundations that underpin trainers’ ethical reasoning (69). Trainers with stronger rights- or duty-based orientations—aligned with the moral foundations of *Care* and *Fairness*—are likely to prioritize the avoidance of harm and the protection of canine autonomy, which may correspond with greater reluctance toward punishment-based techniques. Conversely, individuals who emphasize moral foundations such as *Authority* or *Loyalty* may justify limited aversive use when it appears to advance broader goods such as public safety, owner control, or long-term welfare stability. Identifying the moral foundations associated with different approaches to training would not only clarify how these differences manifest but also provide a conceptual framework through which shared understanding may be fostered (70).

The methodological pluralism evident across interviews—particularly among mixed methods trainers—highlighted both the field’s diversity and its need for a more coherent ethical decision-making model. While such pluralism can be a strength, reflecting adaptability and resistance to a one-framework-fits-all mentality, the absence of shared ethical parameters risks inconsistency and uneven welfare standards. Although the LIMA hierarchy and Fernández’s (71) Least Inhibitive, Functionally Effective (LIFE) model provide useful starting points, both remain primarily focused on the immediate dog–handler interaction. A next-generation framework could integrate these descriptive insights from moral psychology into a more robust decision-making model—one that preserves contextual flexibility while guiding trainers to weigh broader ethical and situational factors such as owners’ resources, time, and physical capacities, alongside short- and long-term welfare outcomes and the potential consequences of inaction, including relinquishment or euthanasia.

#### 4.6.3 Promoting professional cohesion and ethical practice

To strengthen common ground and enhance the practical relevance of future research, we recommend integrating adversarial collaboration (72) into a community-based participatory research framework (73). This hybrid model would engage trainers representing divergent methodological orientations as co-designers throughout all research phases, promoting shared decision-making and mutual accountability in study design and interpretation. Such collaborations could facilitate the co-creation of more comprehensive ethical frameworks while generating richer, more inclusive evidence. Evaluating how these cross-engagement initiatives influence dialogue, intergroup attitudes, and method adoption could yield actionable strategies for advancing professional cohesion. Ultimately, embedding collaboration and reflexivity within research practice has the potential to deepen professional discourse, strengthen trainer–canine relationships, and provide clearer, more consistent ethical and practical guidance to practitioners and the public alike.

## Data Availability

The data supporting this study were largely derived from lengthy qualitative interviews that were transcribed and coded for analysis. Due to the confidential nature of the data and the lack of a standardized format for public sharing, these data are not publicly available. Researchers with specific inquiries may contact the corresponding author to discuss potential access on a case-by-case basis, subject to ethical and confidentiality considerations.

## Footnotes

1 Dogs given more rewards were generally less excitable or aggressive, despite a weak positive link between excitability and reward-based training for unwanted behavior.

2 Zippia aggregates data from federal sources, job postings, and company profiles, analyzed with proprietary algorithms; its accuracy cannot be independently verified.
