# Correction: Professional dog trainers' perspectives on training methods: ethical and evidentiary insights

**DOI:** 10.3389/fvets.2026.1827578

**Published:** 2026-04-16

**Authors:** Jamie L. DeLeeuw, Todd J. Williams

**Affiliations:** 1 Community Research Plus, Grand Rapids, MI, United States; 2 Psychology Department, Grand Valley State University, Allendale, MI, United States

**Keywords:** animal welfare, applied ethics, aversive methods, dog training, epistemology, positive reinforcement, professional regulation, qualitative research

There was a mistake in Table 2 as published. The incorrect value of 1 (6) was listed for Competitive obedience/rally for Positive reinforcement trainers. The corrected Table 2 appears below.

**Table 2:** Demographic and professional characteristics of dog trainers, overall and by training approach.

	Total trainers	Mixed methods trainers	Positive reinforcement trainers
Age	(*N* = 35)	(*n* = 18)	(*n* = 17)
Mean	47	46	48
Median	50	50	50
Range	25–67	25–63	25–67
Gender identity	*N* (%)	*n* (%)	*n* (%)
Women	23 (66)	11 (61)	12 (71)
Men	12 (34)	7 (39)	5 (29)
Region of residence	*N* (%)	*n* (%)	*n* (%)
West	11 (31)	6 (33)	5 (29)
Midwest	6 (17)	6 (33)	0 (0)
Northeast	7 (20)	2 (11)	5 (29)
South	11 (31)	4 (22)	7 (41)
Weekly training hours	*N* (%)	*n* (%)	*n* (%)
< 20	9 (26)	2 (11)	7 (41)
≥20	26 (74)	16 (89)	10 (59)
Years in occupation	*N* (%)	*n* (%)	*n* (%)
2– < 5	4 (11)	2 (11)	2 (12)
5– < 15	16 (46)	7 (39)	9 (53)
15– < 25	12 (34)	6 (33)	6 (35)
≥25	3 (9)	3 (17)	0 (0)
Training	*N* (%)	*n* (%)	*n* (%)
Behavioral	31 (89)	17 (94)	14 (82)
Obedience	16 (46)	9 (50)	7 (41)
Service and assistance	6 (17)	4 (22)	2 (12)
Track and scent	2 (6)	1 (6)	1 (6)
Protection and guard	2 (6)	2 (11)	0 (0)
Agility	1 (3)	0 (0)	1 (6)
Performance	1 (3)	0 (0)	1 (6)
Conformation	1 (3)	0 (0)	1 (6)
Therapy	1 (3)	1 (6)	0 (0)
Competitive obedience/rally	1 (3)	1 (6)	0 (0)

A correction has been made to section heading “3.2.2.2. Assumptions of universal sociability” The word “Assumptions” was separated into two words.

In the published article, the conflict of interest statement was erroneously given as “The author(s) declared that this work was conducted in the absence of any commercial or financial relationships that could be construed as a potential conflict of interest.”

The correct conflict of interest statement is:

“Author JD is the owner of Community Research Plus, a sole proprietorship providing consulting services, and received compensation for work on this project through grant funding.

The remaining author(s) declared that this work was conducted in the absence of any commercial or financial relationships that could be construed as a potential conflict of interest.”

The original version of this article has been updated.

