# Supplementary material for: Professional dog trainers’ perspectives on training methods: ethical and evidentiary insights
Source: Front Vet Sci. 2026 Feb 25;13:1744448. doi: 10.3389/fvets.2026.1744448 (PMC12975608; doi:10.3389/fvets.2026.1744448)
Supplement: Supplementary file 1 [file Table_1.DOCX]

Supplementary Material

# 1 Survey

**Welcome to the Dog Trainer Study!**

**Purpose**: Professional dog training is on the rise, yet an academic understanding of this important community is limited. This study explores factors that motivate individuals to become dog trainers, the advantages and hurdles they face in their profession, public misconceptions, the training methods they employ, and the evolution of these methods over time. Additionally, we aim to capture trainers' key insights that can benefit the wider public.

**Procedure**: You will be asked to complete a brief online survey and then invited to participate in an audio interview (via Zoom or phone) if you qualify. The interview will last about 30-45 minutes, depending on the length of your responses. An audio file will be recorded and then transcribed. Additionally, you must be physically located within the United States at the time of study to participate.

**Privacy and Confidentiality:** Your name will not known to anyone other than the small research team. A pseudonym will be used during the interview, and thus saved as such in the transcription. All information collected from you or about you is for the sole purpose of this research study and will be kept confidential.

**Risks:** Electronic data will be collected and/or stored for this research project. As with any use of electronic means to store data, there exists a minimal risk that data could be lost or stolen.

**Potential Benefits to You:** Benefits include being able to share and reflect on your work.

**Potential Benefits to Society:** Little research has been published about professional dog trainers. More awareness of the benefits of dog training and trainers’ challenges, successes, and insights could promote the field and benefit dogs and their caretakers.

**Appreciation:** As a small token of appreciation for your time and expertise, after participating in an interview you will receive a $10 Amazon e-gift card, or you can have it donated to the American Society for the Prevention of Cruelty to Animals.

**Voluntary Participation:** Your participation in this research study is completely voluntary. You do not have to participate. You may quit at any time without any penalty to you.

**Agreement to Participate**: By filling out the survey, you are agreeing to the following:

-The details of this research study have been explained to me, including what I am being asked to do, and the anticipated risks and benefits;

-I am voluntarily agreeing to participate in the described research, may ask questions, and may quit participating at any time without penalty.

**Researcher Contact Information**: This research is being conducted by Todd Williams, Ph.D., Grand Valley State University, and Jamie DeLeeuw, Ph.D. If you have any questions about the study, you may contact willitod@gvsu.edu.

**Rights:** If you have any questions about your rights as a research participant, please contact the Office of Research Compliance & Integrity at Grand Valley State University, 1 Campus Drive, Allendale, MI. Phone: 616-331-3197. Email: rci@gvsu.edu.

This study has been reviewed by the Institutional Review Board at Grand Valley State University (Protocol #24-093-H).

**I agree to participate.**

☐ Yes

☐ No

**How many hours do you work as a dog trainer per week on average, including activities like training dogs, teaching classes, counseling dog owners, and training preparation/follow-up?**

☐ Fewer than 20 hours

☐ 20 hours or more

**How long have you been a professional dog trainer?**

☐ Less than two years

☐ Two years to under five years

☐ Five years to under fifteen years

☐ Fifteen years to under twenty-five years

☐ Twenty-five years or more

**Please indicate your age in years (e.g., 48).**

_________________________

*Please rate the effectiveness of the following training methods. Examples are provided to illustrate the concepts, and are not exhaustive.*

**Classical Conditioning: Learning through association. For example, to help a dog become more comfortable around other dogs, a treat may be given when another dog approaches, regardless of behavior (distinguishing it from reinforcement).**

☐ Not at all effective

☐ Slightly effective

☐ Moderately effective

☐ Very effective

☐ Extremely effective

☐ I don't know

**Positive Reinforcement: Rewards desired behavior with treats, praise, or other pleasant stimuli.**

☐ Not at all effective

☐ Slightly effective

☐ Moderately effective

☐ Very effective

☐ Extremely effective

☐ I don't know

**Positive Punishment: Adds an aversive stimulus such as an alpha roll, shock, or leash jerk, to decrease an undesirable behavior.**

☐ Not at all effective

☐ Slightly effective

☐ Moderately effective

☐ Very effective

☐ Extremely effective

☐ I don't know

**Negative Reinforcement: Removes or reduces an unpleasant stimulus such as physical pressure or a loud noise when a desired behavior is performed.**

☐ Not at all effective

☐ Slightly effective

☐ Moderately effective

☐ Very effective

☐ Extremely effective

☐ I don't know

**Negative Punishment: Removes a rewarding stimulus to decrease an undesirable behavior. For instance, turning away when a dog jumps on you, or discontinuing walking when the dog pulls on the leash.**

☐ Not at all effective

☐ Slightly effective

☐ Moderately effective

☐ Very effective

☐ Extremely effective

☐ I don't know

**What is your gender identity?**

☐ Male

☐ Female

☐ Non-binary / third gender

☐ Prefer not to say

**Interviews will be scheduled over the next 10 days. Will you be physically located in the United States at the time?**

☐ Yes

☐ Maybe

☐ No

*The following questions assess what training methods you currently use or have used in the past. Examples are provided to clarify the concepts, and are not exhaustive. Please answer to the best of your ability.*

**Classical Conditioning is learning through association. For example, to help a dog become more comfortable around other dogs, a treat may be given when another dog approaches, regardless of behavior (distinguishing it from reinforcement). Please select the response that describes your experience as a professional dog trainer.**

☐ Never intentionally used

☐ Currently intentionally use

☐ Previously intentionally used but discontinued use

**Positive Reinforcement rewards desired behavior with treats, praise, or other pleasant stimuli. Please select the response that describes your experience as a professional dog trainer.**

☐ Never intentionally used

☐ Currently intentionally use

☐ Previously intentionally used but discontinued use

**Positive Punishment adds an aversive stimulus such as an alpha roll, shock, or leash jerk, to decrease an undesirable behavior. Please select the response that describes your experience as a professional dog trainer.**

☐ Never intentionally used

☐ Currently intentionally use

☐ Previously intentionally used but discontinued use

**Negative Reinforcement removes or reduces an unpleasant stimulus such as physical pressure or a loud noise when a desired behavior is performed. Please select the response that describes your experience as a professional dog trainer.**

☐ Never intentionally used

☐ Currently intentionally use

☐ Previously intentionally used but discontinued use

**Negative Punishment removes a rewarding stimulus to decrease an undesirable behavior. For instance, turning away when a dog jumps on you, or discontinuing walking when the dog pulls on the leash. Please select the response that describes your experience as a professional dog trainer.**

☐ Never intentionally used

☐ Currently intentionally use

☐ Previously intentionally used but discontinued use

**Please provide dates (MM/DD) and times you are likely to be available for an interview, including weekends. Please also include your time zone or state of residence. For example, 1) 1/15 from 3–5pm Michigan time. 2) 1/20 from 10–11am Eastern time. 3) 1/22–1/26 from 4–7pm Pacific time.**

Entry 1 ________________________________

Entry 2 ________________________________

Entry 3 ________________________________

Entry 4 ________________________________

Entry 5 ________________________________

Entry 6 ________________________________

# 2 Interview Questions: Mixed Methods Trainers

## Background and Inspiration

1. What motivated you to pursue a career as a dog trainer, and how long have you been in the profession?
2. Are there any individuals or organizations that inspire your work as a trainer?

**Professional Role and Daily Activities**

1. What is your role within your company?
   1. What are your primary responsibilities in this role?
   2. How many other trainers, if any, are employed at your organization?
2. Please describe a typical workday.
   1. What types of dogs do you train?

## Dog Owners

1. How do you involve dog owners in the training process?
2. What do you consider the biggest mistake dog owners make in training?

## Training Philosophy and Methods

1. What guiding philosophies or methods do you follow in your dog training practices?
2. Please provide examples of the results you aim to achieve through your training?
3. Please describe the techniques and equipment you typically use in training and why do you choose to employ these?
4. [If not addressed previously]: You mentioned using positive punishment or negative reinforcement on the survey. What do you use and when do you use it?
5. [If self-employed or owners, skip the next 3 and instead ask, Can your trainers pick their methods, techniques, and equipment, or do you have requirements or restrictions?]
6. Does your organization require you to use specific training methods, techniques, or equipment? If so, please specify.
7. Does your organization prohibit you from using specific training methods, techniques, or equipment? If so, please specify.
8. Are there any training methods, techniques, or equipment that you would prefer to use or avoid that go against organizational policy? If so, please specify.
9. Within your field, are there any training methods, techniques or equipment that you believe should not be used in training? If so, why?

## Comfort and Emotional Well-Being of Dogs

1. How important is the emotional well-being of dogs during training sessions? Please explain.
2. Do you take any specific measures to ensure the emotional well-being of dogs during training sessions?

## Challenges

1. What is the biggest challenge you face in your role as a dog trainer?
2. What aspects of dog training do you believe the public should have a better understanding of? Are there prevalent misconceptions you'd like to address?
3. Some of my interviews have been concerned with trainers not properly understanding or handling dogs. What are your thoughts on regulation of the industry? If you’re in favor, what would this ideally look like?
4. Some trainers describe certain dog behaviors as 'disrespectful,' while others argue that dogs lack the capacity to understand human social concepts like 'disrespect.' What is your perspective?

## Tailoring to Individual Needs and Punishment

Please rate your level of agreement with the following statements on a 1-5 scale where 1 = Strongly disagree, 2 =Moderately disagree, 3 = Neither disagree nor agree, 4 =Moderately agree, 5 = Strongly agree.

1. I adapt my training method and techniques to the specific dog I am working with.
   1. [For those who agree] Please provide an example
   2. [For those who are neutral or disagree] Please explain.
2. There are certain dog behaviors that are **best** modified through positive punishment or negative reinforcement. Positive punishment entails adding an aversive stimulus to decrease an undesirable behavior and negative reinforcement entails removing or reducing an unpleasant stimulus to encourage a desired behavior.
   1. [For those who agree] Please provide an example.
   2. [For those who are neutral or disagree] Please explain.
3. There are certain dogs that respond better to positive punishment or negative reinforcement than other dogs.
   1. [For those who agree] Please provide an example.
   2. [For those who are neutral or disagree] Please explain.
4. There are certain dog breeds that respond better to positive punishment or negative reinforcement than others.
   1. [For those who agree] Please provide an example
   2. [For those who are neutral or disagree] Please explain.

## Personal and Training Evolution

1. Have your training methods or equipment preferences changed over time? If so, what led to these changes?
2. Some trainers have mentioned dogs avoiding them or being fearful when they use positive punishment or negative reinforcement. Have you had this experience?
3. Is it possible to be effective in training without the usage of positive punishment and negative reinforcement?
4. In your dog training practice, can you describe a scenario or situation where you would reevaluate your training methods? What factors do you consider in making this decision?
5. Have there been any training situations where you wished you could change your actions or have regrets? Please explain.

**Additions that some participants received if there was extra time:**

1. You mentioned that purely positive training has limitations. Where does this approach go wrong?
2. Can you tell me about your experience working with veterinarians to determine possible underlying health-related causes of behavioral issues?
3. Have you experienced compassion fatigue from your work? Compassion fatigue typically describes the physical, emotional, and psychological impact of helping others.
4. To what extent do you think there is a negative stigma attached to using positive punishment and negative reinforcement, from 1, not at all, to 5, an extreme amount?

## Concluding Questions

1. What has been your most rewarding moment or achievement as a trainer?
2. Putting dog training aside, what traits or qualities do you generally value most in other people?
3. Can you share some personal interests or hobbies you enjoy in your free time?
4. Lastly, is there anything else you would like me to know or that you thought I should ask?

# 3 Interview Questions: Positive Reinforcement Trainers

# Background and Inspiration

1. What motivated you to pursue a career as a dog trainer, and how long have you been in the profession?
2. Are there any individuals or organizations that inspire your work as a trainer?

**Professional Role and Daily Activities**

1. What is your role within your company?
   1. What are your primary responsibilities in this role?
   2. How many other trainers, if any, are employed at your organization?
2. Please describe a typical workday.
   1. What types of dogs do you train?

## Dog Owners

1. How do you involve dog owners in the training process?
2. What do you consider the biggest mistake dog owners make in training?

## Training Philosophy and Methods

1. What guiding philosophies or methods do you follow in your dog training practices?
2. Please provide examples of the results you aim to achieve through your training?
3. Please describe the techniques and equipment you typically use in training and why do you choose to employ these?
4. [If self-employed or an owner, skip the next 3 and instead ask, Can your trainers pick their methods, techniques, and equipment, or do you have requirements or restrictions?]
5. Does your organization require you to use specific training methods, techniques, or equipment? If so, please specify.
6. Does your organization prohibit you from using specific training methods, techniques, or equipment? If so, please specify.
7. Are there any training methods, techniques, or equipment that you would prefer to use or avoid that go against organizational policy? If so, please specify.
8. Within your field, are there any training methods, techniques or equipment that you believe should not be used in training? If so, why?

## Comfort and Emotional Well-Being of Dogs

1. How important is the emotional well-being of dogs during training sessions? Please explain.
2. Do you take any specific measures to ensure the emotional well-being of dogs during training sessions?

## Challenges

1. What is the biggest challenge you face in your role as a dog trainer?
2. What aspects of dog training do you believe the public should have a better understanding of? Are there prevalent misconceptions you'd like to address?
3. Some of my interviews have been concerned with trainers not properly understanding or handling dogs. What are your thoughts on regulation of the industry? If you’re in favor, what would this ideally look like?
4. Some trainers describe certain dog behaviors as 'disrespectful,' while others argue that dogs lack the capacity to understand human social concepts like 'disrespect.' What is your perspective?

## Tailoring to Individual Needs and Punishment

Please rate your level of agreement with the following statements on a 1-5 scale where 1 = Strongly disagree, 2 =Moderately disagree, 3 = Neither disagree nor agree, 4 =Moderately agree, 5 = Strongly agree.

1. I adapt my training method and techniques to the specific dog I am working with.
   1. [For those who agree] Please provide an example
   2. [For those who are neutral or disagree] Please explain.
2. There are certain dog behaviors that are **best** modified through positive punishment or negative reinforcement. Positive punishment entails adding an aversive stimulus to decrease an undesirable behavior and negative reinforcement entails removing or reducing an unpleasant stimulus to encourage a desired behavior.
   1. [For those who agree] Please provide an example.
   2. [For those who are neutral or disagree] Please explain.
3. There are certain dogs that respond better to positive punishment or negative reinforcement than other dogs.
   1. [For those who agree] Please provide an example.
   2. [For those who are neutral or disagree] Please explain.
4. There are certain dog breeds that respond better to positive punishment or negative reinforcement than others.
   1. [For those who agree] Please provide an example
   2. [For those who are neutral or disagree] Please explain.

## Personal and Training Evolution

1. You previously indicated that your training methods included positive punishment or negative reinforcement in the past. Which techniques and equipment did you use?
2. Did you experience any unease or discomfort when using these methods? Please specify.
3. What led you to stop or reduce your usage of positive punishment or negative reinforcement?

a. Was it a gradual or sudden process? Please explain.

1. Did you experience any challenges once you decided to no longer use positive punishment or negative reinforcement. If so, please explain.
2. Were there any benefits from eliminating your usage of positive punishment or negative reinforcement? If so, please explain.
3. Looking into the future with your dog training practice, can you describe a scenario or situation where you would reevaluate your training methods? What factors do you consider in making this decision?
4. Have there been any training situations where you wished you could change your actions or have regrets? Please explain.

**Additions that some participants received if there was extra time:**

1. Why do you think some trainers use positive punishment or negative reinforcement?
2. Can you tell me about your experience working with veterinarians to determine possible underlying health-related causes of behavioral issues?
3. Have you experienced compassion fatigue from your work? Compassion fatigue typically describes the physical, emotional, and psychological impact of helping others.
4. To what extent do you think there is a negative stigma attached to using positive punishment and negative reinforcement, from 1, not at all, to 5, an extreme amount?

## Concluding Questions

1. What has been your most rewarding moment or achievement as a trainer?
2. Putting dog training aside, what traits or qualities do you generally value most in other people?
3. Can you share some personal interests or hobbies you enjoy in your free time?
4. Lastly, is there anything else you would like me to know or that you thought I should ask?
